# Understanding the behavioral intentions of MaaS during public health events

**DOI:** 10.3389/fpsyg.2024.1517783

**Published:** 2025-01-08

**Authors:** Hang Luo, Shoufeng Ma, Junfang Tian, Hongming Dong

**Affiliations:** ^1^Institute of Systems Engineering, College of Management and Economics, Tianjin University, Tianjin, China; ^2^Laboratory of Computation and Analytics of Complex Management Systems (CACMS), Tianjin University, Tianjin, China; ^3^School of International Education, Tianjin University, Tianjin, China

**Keywords:** mobility as a service, public health emergencies, UTAUT model, ambiguity tolerance, behavioral intention

## Abstract

**Introduction:**

Mobility as a Service (MaaS) integrates various modes of transportation, provides personalized travel services for travelers, and improves the efficiency of traditional travel modes. To examine the mechanisms underlying the impact of sudden public health events on the behavioral intentions to use MaaS and provide theoretical support for the sustainable development of MaaS, this research investigates the Beijing MaaS program as a case.

**Methods:**

A total of 630 questionnaires were collected. Theoretical model, sourced from the Unified Theory of Acceptance and Use of Technology (UTAUT) model, is employed to elucidate the influence of six variables—performance expectancy, effort expectancy, social influence, ambiguity tolerance, perceived health threat, and policy cognition—on the behavioral intentions of MaaS.

**Results:**

The results show that three variables from the UTAUT positively impact behavioral intention and that there is a significant mediating effect of policy cognition on the relationship between these variables and the intention to use. Travelers with a high level of ambiguity tolerance are more inclined to use MaaS, while the perceived health threat reduces the intentions.

**Discussion:**

Multigroup analysis revealed differences in effort expectancy, ambiguity Toleance, and perceived health threat among the various groups. The research findings may provide theoretical guidance and empirical evidence for the promotion strategies of MaaS and for the formulation of related policies.

## 1 Introduction

The accelerated process of global urbanization and the rapid growth in transportation demand have posed substantial challenges to traditional transportation services. To address these issues, Mobility as a Service (MaaS) has emerged as an innovative framework that integrates diverse transportation modes, including public transportation, taxis, shared bicycles, and ride-sharing services, into a unified platform supported by information technology, offering one-stop services and prepayment discount packages (Hietanen, 2014; Smith and Hensher, 2020). However, significant public health events, such as the COVID-19 pandemic, have disrupted the early promotion of MaaS due to personal travel restrictions, interruptions in transportation services, and heightened risks of virus transmission during transfers or shared rides (Kapser et al., 2021; Mulley et al., 2023). These challenges underscore the urgency of clarifying the mechanisms through which public health events influence travelers’ behavioral intentions to adopt MaaS (Enoch and Potter, 2023; Hensher et al., 2021; Sokolowska et al., 2021).

In recent years, research on the behavioral intention to use MaaS has primarily focused on the influence of psychological factors (Alonso-González et al., 2020; Fioreze et al., 2019; Ho et al., 2018; Schikofsky et al., 2020; Strömberg et al., 2018), demographic attributes (Lopez-Carreiro et al., 2021), and MaaS package designs (Bahamonde-Birke et al., 2024; Brezovec and Hampl, 2021; Hensher and Mulley, 2021; Kriswardhana and Esztergár-Kiss, 2023; Vij et al., 2020). Additionally, some studies have noted that travelers using MaaS receive a greater volume of information compared to those using individual transportation services (Bahamonde-Birke et al., 2024; Hensher, 2022; Orozco-Fontalvo and Moura, 2023). However, there is a scarcity of in-depth analyses regarding the intrinsic impact mechanisms by which the increased volume of information—including ambiguous information—affects travelers’ behavioral intentions. Ambiguous information, defined as unfamiliar, complex, or inconsistent information (Furnham and Ribchester, 1995), is a prominent factor in MaaS package selection and route recommendations (Lauriola et al., 2007). Such information may induce anxiety and uneasiness among travelers, though individuals with high ambiguity tolerance are less susceptible to negative emotions in such contexts (McLain, 2009; Xu et al., 2016). Ambiguity tolerance reflects individuals’ perceptual and cognitive processing modes when confronted with unfamiliar, complex, or inconsistent cues (Furnham and Ribchester, 1995), and it has been shown to influence decision-making behavior (Tian and Li, 2015), travel choices, and travelers’ willingness to participate in ride-sharing (Zhang and Liu, 2022). Moreover, individuals with lower ambiguity tolerance are more likely to exhibit avoidance behavior (Lauriola and Levin, 2001). Despite these insights, it remains unclear whether and how ambiguity tolerance affects travelers’ adoption of MaaS, necessitating further investigation into its underlying mechanisms.

The influence of perceived health threats on travel behavior has also garnered increasing scholarly attention, particularly in the context of public health events. Perceived health threats refer to individuals’ subjective evaluations of potential risks to their health (Maiman and Becker, 1974) and are influenced by travelers’ heightened health concerns during public health crises (Dong et al., 2021). Research has revealed that individuals are more inclined to use private vehicles during public health events to minimize exposure to the risk of virus transmission associated with public or shared transportation modes (Upadhyay et al., 2022). This behavioral trend is especially pronounced for medium travel distances (6–12 km), where only 17.45% of travelers opt for non-private modes of transportation (Luan et al., 2021). Moreover, individuals perceiving significant health threats are less likely to adopt travel modes involving ride-sharing (Zhang and Liu, 2022). As MaaS inherently integrates multiple transportation modes, it becomes essential to explore how perceived health threats shape travelers’ behavioral intentions toward adopting MaaS.

In addition to psychological factors, government-implemented travel management policies during public health crises have played a crucial role in influencing travel behavior by mitigating virus transmission risks and promoting safe travel practices (Hensher, 2020). Travel management policies exert multifaceted influences on individual behavior, which are contingent upon the level of individual policy cognition (Luo M. et al., 2022; Luo P. et al., 2022). Policy cognition, defined as the depth of individuals’ understanding of policy content (Xiong and Wang, 2020), has been shown to influence policy compliance. For example, family factors can lead to significant variations in individuals’ perceptions of policies (Wang et al., 2020). Existing research on policy cognition in the transportation domain has predominantly focused on restrictive measures, such as motor vehicle tail-number restrictions (Luo M. et al., 2022; Luo P. et al., 2022). However, evidence regarding whether and how individual policy cognition directly affects travel behavior remains limited. This research anticipates that individuals, upon receiving information on transportation management policies and forming policy cognition, may adjust their preexisting travel behavior (Potter, 2012). Consequently, this study aims to define the mechanisms by which policy cognition influences the adoption of MaaS.

To address these research gaps, this study integrates ambiguity tolerance, perceived health threats, and policy cognition into the Unified Theory of Acceptance and Use of Technology (UTAUT) model to comprehensively examine their impact mechanisms on travelers’ adoption of MaaS. The UTAUT model, which builds upon the Technology Acceptance Model (TAM), has been widely employed in understanding travelers’ behavioral intentions due to its robust explanatory power in technology adoption research (Adnan et al., 2018; Kapser et al., 2021; Tran et al., 2019; van’t Veer et al., 2023). In the original UTAUT model, variables such as performance expectancy, effort expectancy, and social influence are considered key determinants of behavioral intentions (Venkatesh et al., 2003). This research extends the UTAUT framework by incorporating the aforementioned psychological and contextual factors, aiming to (1) clarify the role of ambiguous information in influencing travelers’ utilization of MaaS, (2) analyze the mechanisms through which perceived health threats mediate the relationship between public health events and MaaS adoption, and (3) investigate how policy cognition shapes travelers’ behavioral intentions.

This study is structured as follows: first, a comprehensive review of relevant literature, the establishment of a theoretical framework, and the formulation of research hypotheses; second, the design of a research questionnaire and data collection; third, the analysis of the collected data to derive research findings and conclusions; and finally, the formulation of managerial recommendations and policy suggestions based on the findings to support the sustainable adoption of MaaS.

## 2 Theoretical background and hypotheses

### 2.1 Performance expectancy

The literature has shown that performance expectancy is a crucial factor influencing users’ adoption of new technologies (van’t Veer et al., 2023; Zhou et al., 2019). Performance expectancy is the extent to which individuals believe that adopting new technology can significantly enhance their job performance (Venkatesh et al., 2003). In the context of MaaS, performance expectancy is users’ desire to improve their travel efficiency by using MaaS. This expectation is built on the understanding and trust of travelers in the convenience, flexibility, and timeliness provided by MaaS. This construct plays a pivotal role in shaping users’ behavioral intentions to adopt MaaS, as it directly relates to their perceived benefits of employing the service to improve their travel experience. By emphasizing and addressing performance expectancy, policymakers and service providers can ensure that MaaS meets user expectations, thereby increasing its acceptance and adoption. Adnan et al. (2018) proposed in their research on autonomous driving that when users perceive that the product they are using can provide convenience and time savings, there is a significant increase in users’ intention to use. According to previous research, this research posits that performance expectancy will positively influence the intention to use MaaS (van’t Veer et al., 2023). In particular, during public health events, the safety and accessibility of travel modes are crucial indicators for assessing travel performance. Based on this, the following hypothesis is proposed:

*Hypothesis 1*: Performance expectancy positively influences the behavioral intention to adopt MaaS.

### 2.2 Effort expectancy

Effort expectancy is the ease with which a new technology can be used and adopted (Venkatesh et al., 2003). For innovative mobility services such as MaaS, the ease of design and operation is a crucial factor in its widespread adoption. In this research, effort expectancy is defined as the level of effort users need to exert when using MaaS services. Existing research have confirmed that travel modes with higher usability are more likely to enhance travelers’ behavioral intentions (Mohammed et al., 2020). However, other research has shown that effort expectancy is not a significant factor influencing behavioral intentions (Madigan et al., 2017). This research posits that, compared to discrete mobility services, MaaS integrates existing transportation services, with usage methods and rules closely resembling those of current services (Ye et al., 2020). Travelers can access various modes of transportation through unified MaaS (Cooper and Vanoutrive, 2022), eliminating the need for repetitive smartphone interactions during the usage process (Dadashzadeh et al., 2022). From this, it can be inferred that the usability of MaaS may influence travelers’ intentions to use the service. Based on this, the following hypothesis is proposed:

*Hypothesis 2*: Effort expectancy positively influences the behavioral intention to adopt MaaS.

### 2.3 Social influence

Furthermore, numerous research have corroborated the impact of social influence on the use intentions of users (van’t Veer et al., 2023; Ye et al., 2020). This is primarily attributed to individuals who adjust their thoughts based on the reactions of friends or peers (Wang et al., 2021). In the context of this research, social influence is predominant the influence on users’ choice to adopt MaaS due to the usage behavior and recommendations of significant others in their surroundings, such as family and friends. Research has shown that the perspectives of key figures and societal assessments of products are crucial factors influencing user adoption (Zhou et al., 2019). This research posits that social influence positively affects user behavioral intentions when considering the adoption of MaaS (Tran et al., 2019). During significant public health events, the behaviors of others are more likely to become a crucial factor in users’ assessments of whether to opt for MaaS (Kapser et al., 2021). Specifically, important interpersonal relationships, such as family members, during such events significantly influence travelers’ intentions to use MaaS, with social influence playing a substantial role (Jansson et al., 2017). Based on this, the following hypothesis is proposed:

*Hypothesis 3*: Social influence positively influences the behavioral intention to adopt MaaS.

### 2.4 Ambiguity tolerance

Individuals with different levels of ambiguity tolerance can exhibit varying emotional responses to ambiguous situations. Individuals with high ambiguity tolerance tend to find impending ambiguous situations interesting or challenging (Furnham and Ribchester, 1995), while individuals with low ambiguity tolerance often experience feelings of unease, anxiety, or tension in such circumstances (Hazen et al., 2012). As a novel form of integrated mobility, MaaS consolidates commonly used modes of transportation for the public while also accommodating diverse user profiles engaging in various types of travel. However, this integration approach itself may introduce ambiguous situations for users. For example, for travelers who are accustomed to a single mode of transportation or to using private cars, previous experiences may not provide insight into the actual experiential aspects of integrated mobility. Within such ambiguous contexts, there is potential to diminish the overall travel experience for users, consequently leading to a reduced willingness to adopt MaaS (Hazen et al., 2012). Research on employees’ proactive behaviors revealed that employees with a high level of ambiguity tolerance are more likely to engage in challenging proactive behaviors when they perceive uncertainty and risk (Tian and Li, 2015). Similarly, this research posits that users with high ambiguity tolerance are more inclined to make early attempts at using MaaS, thereby enhancing their intention to use MaaS. Based on this, the following hypothesis is proposed:

*Hypothesis 4*: Ambiguity tolerance positively influences the behavioral intention to adopt MaaS.

### 2.5 Perceived health threat

During public health events, public health has become a potential concern. In this research, our focus is on analyzing the impact of perceived health threat on users’ intentions to use MaaS. Perceived health threats depend on users’ perceptions of potential threats to their health that may arise during the use of MaaS. Research on the participation of diabetic patients in physical activity revealed a significant relationship between the perceived severity of symptoms and exercise behavior (Alaiad et al., 2019). Research assessing users’ adoption of health products to monitor their health status found that as the perceived health threat increases, there is a significant increase in users’ intentions to adopt health products (Beh et al., 2021). Consequently, perceived health threats are identified as crucial factors influencing consumers’ choice of specific products or services. In MaaS, the service itself includes subways, busses, and ridesharing, which involve shared mobility. These shared mobility practices contribute to an increased perception of health threats among users, consequently reducing their willingness to adopt MaaS (Kapser et al., 2021).

*Hypothesis 5a*: Perceived health threats negatively influence the behavioral intention to adopt MaaS.

In research on urban residents’ awareness of protection in the context of the public health events, some scholars posit that individual protective awareness is positively influenced by altruism. This is attributed to the fact that serving as a medium for virus transmission can subject individuals to social pressures of irresponsibility toward others and themselves (Wang et al., 2021). Social influence, in turn, can significantly mitigate the negative impact on individuals and, consequently, enhance their protective awareness. Therefore, this research posits that as the perceived health threat increases, users contemplating the adoption of MaaS will place greater emphasis on others’ usage of MaaS. This contributes to reducing the social pressure that users may experience during the use of MaaS due to the increased risk of virus transmission.

*Hypothesis 5b*: Social influence mediates the relationship between perceived health threat and behavioral intention.

Furthermore, research on the proactive behaviors of employees has shown that employees with lower ambiguity tolerance are more susceptible to emotional influences such as anxiety and stress. In contrast, employees with greater ambiguity tolerance demonstrate a greater ability to embrace risk (Tian and Li, 2015). Therefore, this research posits that ambiguity tolerance mediates the relationship between perceived health threat and behavioral intention. Specifically, as the perceived severity of health threats increases, travelers with lower ambiguity tolerance are more susceptible to anxiety and stress. Moreover, they find it more challenging to embrace the ambiguous situations inherent in the use of MaaS, consequently leading to a diminished intention to use MaaS.

*Hypothesis 5c*: Ambiguity tolerance mediates the relationship between perceived health threat and behavioral intention.

### 2.6 Policy cognition

Cognition is the internal processing of information by individuals and encompasses processes such as input, storage, and information retrieval (Potter, 2012). Human cognition is typically classified into innate and acquired categories, with innate cognition influenced by factors such as genetics and health, while acquired cognition is subject to various factors. Policy cognition is a prominent form of acquired cognition shaped primarily by factors such as educational background, personal experiences, and the social environment (Potter, 2012). Compared to general cognition, the level of policy cognition more effectively reflects an individual’s cognitive pathways and depth of understanding. This research posits that during public health events, individuals, in response to national calls, tend to pay attention to relevant prevention and control policies before traveling. Understanding such policies often requires individuals to possess a higher level of policy cognition. Users with a higher level of cognition may prioritize factors such as performance expectancy, effort expectancy, and social influence when considering the adoption of MaaS (Daniali et al., 2022). Therefore, it is suggested that users’ policy cognitions may play a mediating role in the impact of the aforementioned factors on the intention to use MaaS.

*Hypothesis 6a*: Performance expectancy mediates the relationship between policy cognition and behavioral intention.

*Hypothesis 6b*: Effort expectancy mediates the relationship between policy cognition and behavioral intention.

*Hypothesis 6c*: Social influence mediates the relationship between policy cognition and behavioral intention.

### 2.7 The moderating effect of age

Regarding the moderating effect of age, previous studies suggest that younger travelers typically exhibit higher performance expectancy for new travel services (Venkatesh et al., 2003). They focus on the convenience, efficiency, and flexibility provided by technology, which strongly influences their intention to use such services. In contrast, older travelers tend to be more hesitant about adopting new technologies and prefer familiar, traditional travel options. This indicates that younger travelers are more likely to benefit from the performance expectancy of new travel services, which significantly shapes their usage intentions (van’t Veer et al., 2023). However, this study argues that for older travelers, when MaaS meets their performance expectancy, it may exert a stronger positive impact on their intention to use. While performance expectancy also influences younger travelers, its effect may not be as pronounced compared to its impact on older travelers.

In terms of effort expectancy, older travelers find it relatively more challenging to learn and adapt to new technologies, often requiring more time and effort to overcome technical barriers (Mohammed et al., 2020). Therefore, their effort expectancy for new technologies tends to be higher, as ease of use can help them reduce learning costs and adaptation difficulties (Ye et al., 2020). In contrast, younger travelers typically possess stronger learning abilities, enabling them to adapt to and master new technologies more effectively. Consequently, although effort expectancy can also influence younger travelers’ intentions to use new technologies, its impact may be relatively smaller. This study posits that when older travelers’ effort expectancy is satisfied, their intention to use such technologies significantly increases.

*Hypothesis 7a:* Age moderates the impact of performance expectancy on the intention to use, with older travelers’ intention to use MaaS being more likely to increase significantly as the levels of these factors rise.

*Hypothesis 7b:* Age moderates the impact of effort expectancy on the intention to use, with older travelers’ intention to use MaaS being more likely to increase significantly as the levels of these factors rise.

### 2.8 Multiple-group SEM

In the research domain of MaaS, scholars have extensively examined the heterogeneity of individuals’ willingness to adopt MaaS based on demographic characteristics. Specifically, some research conducted within the framework of the UTAUT model have revealed the differentiated roles of factors such as gender, marital status, private car ownership, and possession of a driver’s license in shaping individuals’ intentions to adopt MaaS (Matyas and Kamargianni, 2019; Schikofsky et al., 2020; Strömberg et al., 2018). Other research has classified the travel behaviors of travelers, identifying factors such as travel distance and destination as crucial elements that influence individual adoption of MaaS (Lopez-Carreiro et al., 2021).

Given China’s vast population and diverse regional characteristics, this research aims to investigate the mechanisms through which demographic and regional characteristics influence individuals’ intentions to use MaaS. Elucidating the impact of these features on the willingness to use MaaS in the research model contributes to a more comprehensive delineation of potential user profiles. Therefore, this research further analyses the main effects of the research (H1–H7b) and investigates in depth the differentiated impact mechanisms of five demographic characteristics (gender, marital status, driving qualification, travel distance, and travel destination) on the main effects of the first-order model. This serves to enhance the practical significance of this research.

**Figure 1:**
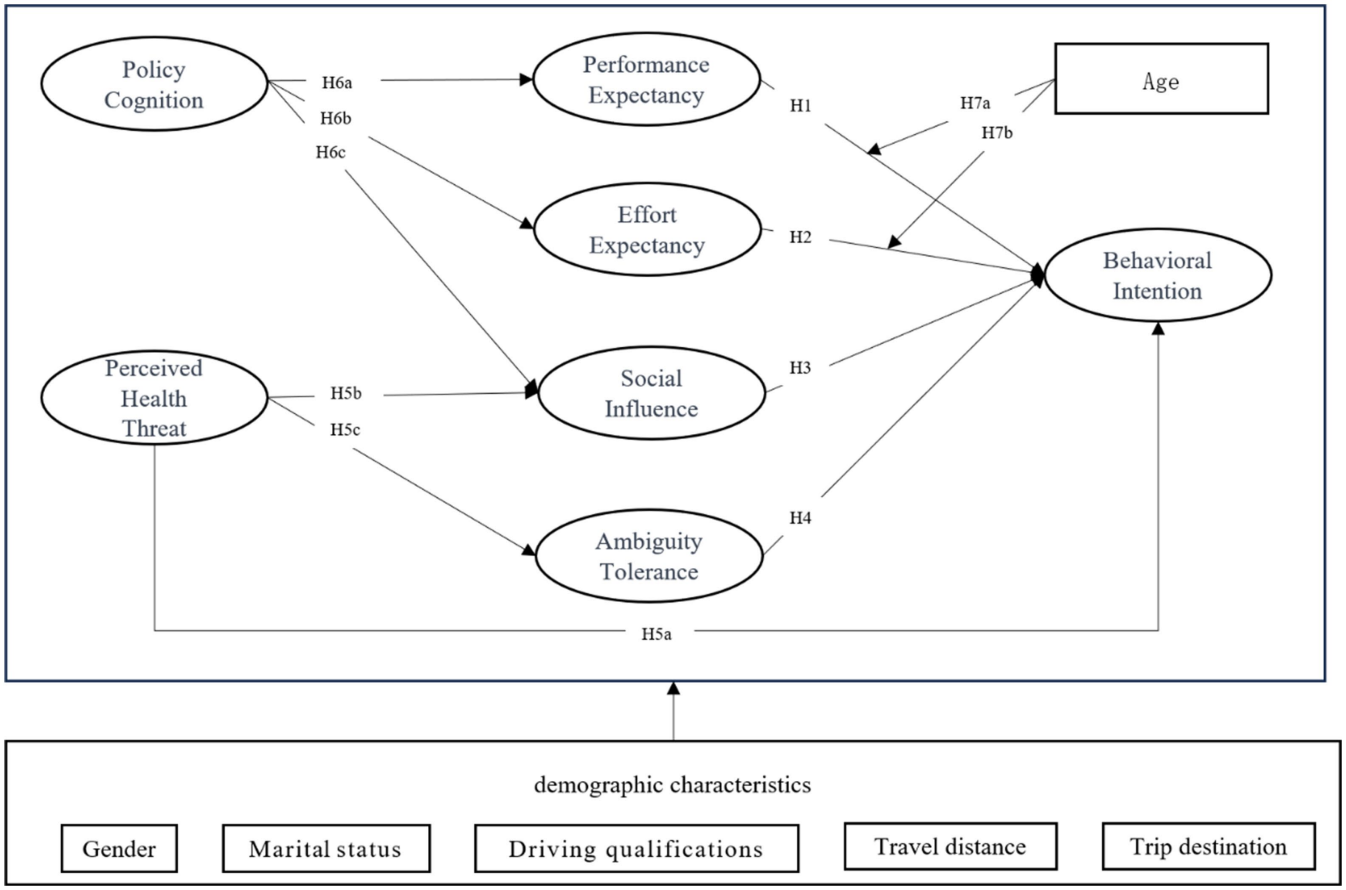
Theoretical framework.

Given the research hypotheses mentioned above, this research developed the theoretical model diagram presented in Figure 1.

## 3 Methodology

### 3.1 Sampling and analytical procedures

This research conducted an online questionnaire survey in Beijing using the WenJuanXing (a data collection website similar to Mturk) platform from July 15 to December 5, 2022. Each participant spent an average of 10 min completing the questionnaire, and upon successful completion without errors or omissions, they received a cash reward of 6 yuan. The choice of Beijing as the survey location was motivated by its status as one of the first cities in China to promote MaaS, ensuring that the samples obtained have a certain representativeness. After the questionnaire service was closed, 703 responses were received. Following a careful screening of invalid responses, a total of 630 valid questionnaires were obtained (Table 1).

**Table 1:** Characteristics of the sample (*N* = 630).

Category	Level	Frequency	Percentage
Gender	Male	265	42.1
	Female	365	57.9
Marital status	Married	427	67.8
	Unmarried	203	32.2
Age	Low (<25)	104	16.5
	Mid (25–45)	490	77.8
	High (>45)	36	5.7
Income	Low (<4000 RMB)	71	11.1
	Mid (4000–8000 RMB)	269	42.7
	High (>8000 RMB)	291	46.2
Education	Junior college student and below	67	10.6
	Undergraduate	452	71.7
	Postgraduate	111	17.7
Household composition	Low (<3 head)	83	13.2
	Mid (3 head)	339	53.8
	High (>3 head)	208	33.0
Destination	Into urban	165	26.2
	Suburban	465	73.8
Trip distance	<10 KM	342	54.3
	> = 10 KM	288	45.7
Private car	Yes	165	26.2
	No	465	73.8
Driving qualifications	With a driver’s license	479	76.0
	Without a driver’s license	151	24.0
Frequency	Low (don’t drive)	152	24.1
	Mid (monthly 1–11 days)	194	30.8
	High (monthly > 11 days)	284	45.1

### 3.2 Survey design and procedure

In this research, to ensure a better understanding of the concept of MaaS among participants, MaaS-related materials were provided (using the Beijing Traffic App as an example) before the participants filled out the questionnaire. Following the reading, the participants sequentially responded to the questionnaire items. The survey instrument was adapted from previous scales and modified according to research needs, and all the elements were rephrased in the context of MaaS to avoid ambiguity among participants, as illustrated in Table 2. The questionnaire used a 7-point Likert scale, where “1” represented “strongly agree,” “4” represented neutral, and “7” represented “strongly disagree.” As presented in Table 3.

**Table 2:** Measurement scales.

Construct	Item	Source
Performance expectancy	PE1: I hope MaaS can optimize travel time.	Venkatesh et al. (2012)
	PE2: I hope MaaS to be more convenient than individual modes of transportation.	
	PE3: I hope to have access to transportation service information anytime, anywhere.	
Effort expectancy	EE1: I comprehend the concept of MaaS.	Venkatesh et al. (2012)
	EE2: I am open to adopting the MaaS.	
	EE3: I find it easy to learn and use MaaS.	
Social influence	SI1: If everyone adopts MaaS, then I am also willing to utilize it.	Venkatesh et al. (2012)
	SI2: If people in the surrounding environment express praise and support for the use of MaaS, then I am also willing to embrace its adoption.	
	SI3: If the media provides favorable assessments of MaaS, then I am inclined to adopt it as well.	
Ambiguity tolerance	AT1: When utilizing MaaS, I am tolerant of situations where the transfer mode is ambiguous.	Zhang and Liu (2022)
	AT2: I enjoy addressing the ambiguous and intricate issues that arise when utilizing MaaS.	
	AT3: I prefer encountering some degree of ambiguity when using MaaS.	
Policy cognition	PC1: I am well-versed in the travel control and management policies implemented during the pandemic period.	Luo M. et al. (2022), Luo P. et al. (2022), and Xu et al. (2016)
	PC2: I am aware of the regulations and measures regarding travel-related epidemic prevention and control policies in the current location during the pandemic period.	
	PC3: I have a profound understanding of the management policies and details related to travel during the pandemic.	
Perceived health threat	PHT1: I believe that contracting COVID-19 during the utilization of MaaS is undesirable.	Alaiad et al. (2019)
	PHT2: For both myself and my family, contracting COVID-19 during the use of MaaS is a distressing experience.	
	PHT3: If I or my family were to contract COVID-19 while utilizing MaaS, we would experience fear in response	
Behavioral intention	BI1: I am highly interested in MaaS.	Venkatesh et al. (2012)
	BI2: In my future daily life, I will endeavor to utilize MaaS.	
	BI3: I plan to utilize MaaS for future transportation needs.	

**Table 3:** Measurement model assessment results.

Construct	Item	Loading	CR	AVE	Mean	SD	Var
Performance expectancy	PE1	0.697	0.786	0.551	3.607	1.233	1.521
	PE2	0.814					
	PE3	0.701					
Effort expectancy	EE1	0.721	0.783	0.547	2.252	0.940	0.883
	EE2	0.785					
	EE3	0.719					
Social influence	SI1	0.815	0.834	0.626	1.907	0.685	0.469
	SI2	0.791					
	SI3	0.766					
Ambiguity tolerance	AT1	0.752	0.805	0.580	2.002	0.677	0.458
	AT2	0.804					
	AT3	0.727					
Policy cognition	PC1	0.817	0.868	0.687	2.176	0.828	0.685
	PC2	0.970					
	PC3	0.769					
Perceived health threat	PHT1	0.853	0.872	0.696	1.974	0.756	0.571
	PHT2	0.854					
	PHT3	0.777					
Behavioral intention	BI1	0.810	0.850	0.653	2.005	0.753	0.567
	BI2	0.815					
	BI3	0.799					

Loading: standardized factor loading; AVE, average variance extracted; CR, composite reliability; SD, standard deviation; Var, variance.

This research used a confirmation factor analysis to assess the reliability and validity of the constructed model. The reliability and internal consistency of the scales were measured using Cronbach’s alpha, and the results were required to be greater than 0.7.

An examination of convergent validity provides insight into the interrelatedness among items within the same variable. In this examination, the emphasis is placed on analyzing factor loadings (values greater than 0.5 and statistically significant at *p* < 0.05 within the same variable) and the average variance extracted (AVE). The mean values for the constructs range between 1.907 and 3.607, demonstrating a diverse range of participant responses across items. The standard deviation (SD) values, varying from 0.458 to 1.233, indicate moderate variability in the data, reflecting differences in individual perceptions. The variance (Var) values, spanning from 0.469 to 1.521, further illustrate the extent of dispersion in the measurements. As presented in Table 3, the results of the examination confirm that the proposed research model exhibits acceptable convergent validity.

This research uses SPSS 26 and AMOS 26 for the statistical analysis of the data; these analyses include descriptive statistics, reliability and validity analyses, and Structural Equation Modeling (SEM). Following the analytical methods of SEM and bootstrapping, an analysis of the usage intention model of MaaS is conducted based on the UTAUT model.

## 4 Results

### 4.1 Assessment of the measurement model

The current literature suggests that the fit of a research model needs to be validated through multiple indicators. In this research, we used three commonly used indices to assess the fit of the model: the Tucker–Lewis Index (TLI ≥ 0.95), the Comparative Fit Index (CFI ≥ 0.95), and the Root Mean Square Error of Approximation (RMSEA ≤ 0.07). The results indicate that the fit of the model in this research meets established standards. The detailed results are presented in Table 4.

**Table 4:** Model fit of research models.

Index	*χ*^2^/df	RMSEA	TLI	CFI
Standard	<3.00	≤ 0.07	≥ 0.90	≥ 0.90
Result	1.446	0.027	0.986	0.988

Unlike convergent validity, discriminant validity is primarily based on the degree of differentiation between a variable and other variables. The assessment criteria mainly involve examining whether the AVE values of all variables exceed 0.5 while also ensuring that the square root of the AVE is greater than the correlation coefficient. As shown in Table 5, the test results demonstrate that the model has acceptable discriminant validity.

**Table 5:** Results of the discriminant validity test.

	BI	PHT	PC	SI	EE	PE	AT
BI	0.808						
PHT	0.032	0.834					
PC	0.495	0.101	0.829				
SI	0.720	0.176	0.413	0.791			
EE	0.790	0.023	0.462	0.619	0.739		
PE	0.779	0.188	0.409	0.707	0.722	0.742	
AT	0.347	−0.099	0.167	0.258	0.385	0.282	0.762

BI, behavioral intention; PHT, perceived health threat; PC, policy cognition; SI, social influence; EE, effort expectancy; PE, performance expectancy; AT, ambiguity tolerance. The value on the diagonal is the square root of AVE.

### 4.2 Assessment of the structural model

After completing the previous verification step, this research analyzed the fit indices for the SEM. The results indicated a good fit for the SEM (*χ*^2^/df: 3.903; *p* < 0.001; CFI: 0.920; TLI: 0.906; RMSEA: 0.068). The SEM analysis of the model is presented in Table 6. Overall, the three crucial variables of the UTAUT model were found to have significant impacts on the intention to use MaaS. Specifically, traveler performance expectancy (*β* = 0.419, *p* < 0.001), effort expectancy (*β* = 0.470, *p* < 0.001), and social influence (*β* = 0.310, *p* < 0.001) were positively correlated with the intention to use MaaS. Furthermore, traveler ambiguity tolerance positively impacted travelers’ intentions to use MaaS (*β* = 0.043, *p* < 0.05), while perceived health threat significantly negatively influenced travelers’ intentions to use MaaS (*β* = −0.063, *p* < 0.05). Therefore, hypotheses 1, 2, 3, 4, and 5a were confirmed through the examination, consistent with the theoretically expected relationships presented in Table 6.

**Table 6:** Hypothesis testing.

Hypothesis	Paths	*t*	*β*	*p*	Comments
H1	PE→BI	+	0.419	<0.001	Support
H2	EE→BI	+	0.470	<0.001	Support
H3	SI→BI	+	0.310	<0.001	Support
H4	AT→BI	+	0.043	<0.05	Support
H5a	PHT→BI	−	−0.063	<0.05	Support

BI, behavioral intention; PHT, perceived health threat; PC, policy cognition; SI, social influence; EE, effort expectancy; PE, performance expectancy; AT, ambiguity tolerance.

After applying the bootstrap method for 5,000 iterations, the mediating effects of effort expectancy, performance expectancy, social influence, and ambiguity tolerance were obtained, as shown in Table 7. The perception of health threats was found to positively influence individuals’ intentions to use MaaS through social influence (*β* = 0.135, *p* < 0.001, CI: −0.145 to −0.020). However, this influence did not occur through ambiguity tolerance (*β* = −0.084, *p* > 0.05, CI: −0.069 to 0.013). Therefore, H5b was validated, while H5c was not. However, policy cognition significantly and positively influenced the intention to use MaaS through performance expectancy (*β* = 0.327, *p* < 0.001, CI: 0.280–0.458), effort expectancy (*β* = 0.365, *p* < 0.001, CI: 0.331–0.487), and social influence (*β* = 0.392, *p* < 0.001, CI: 0.242–0.420). Consequently, H6a, H6b, and H6c were confirmed.

**Table 7:** Mediation effects of research models.

Hypothesis	Paths	Direct effect	Indirect effect	LBCI	UBCI	*p*
H5b	PHT → SI → BI	−0.024	0.209	−0.145	−0.020	*P* < 0.001
H5c	PHT → AT → BI	0.057	−0.026	−0.069	0.013	*P* > 0.05
H6a	PC → PE → BI	/	0.369	0.280	0.458	*P* < 0.001
H6b	PC → EE → BI	/	0.409	0.331	0.487	*p* < 0.001
H6c	PC → SI → BI	/	0.333	0.242	0.420	*p* < 0.001

BI, behavioral intention; PHT, perceived health threat; PC, policy cognition; SI, social influence; EE, effort expectancy; PE, performance expectancy; AT, ambiguity tolerance. The LBCI and UBCI represent the lower and upper bounds of the 95% confidence interval, respectively.

**Figure 2:**
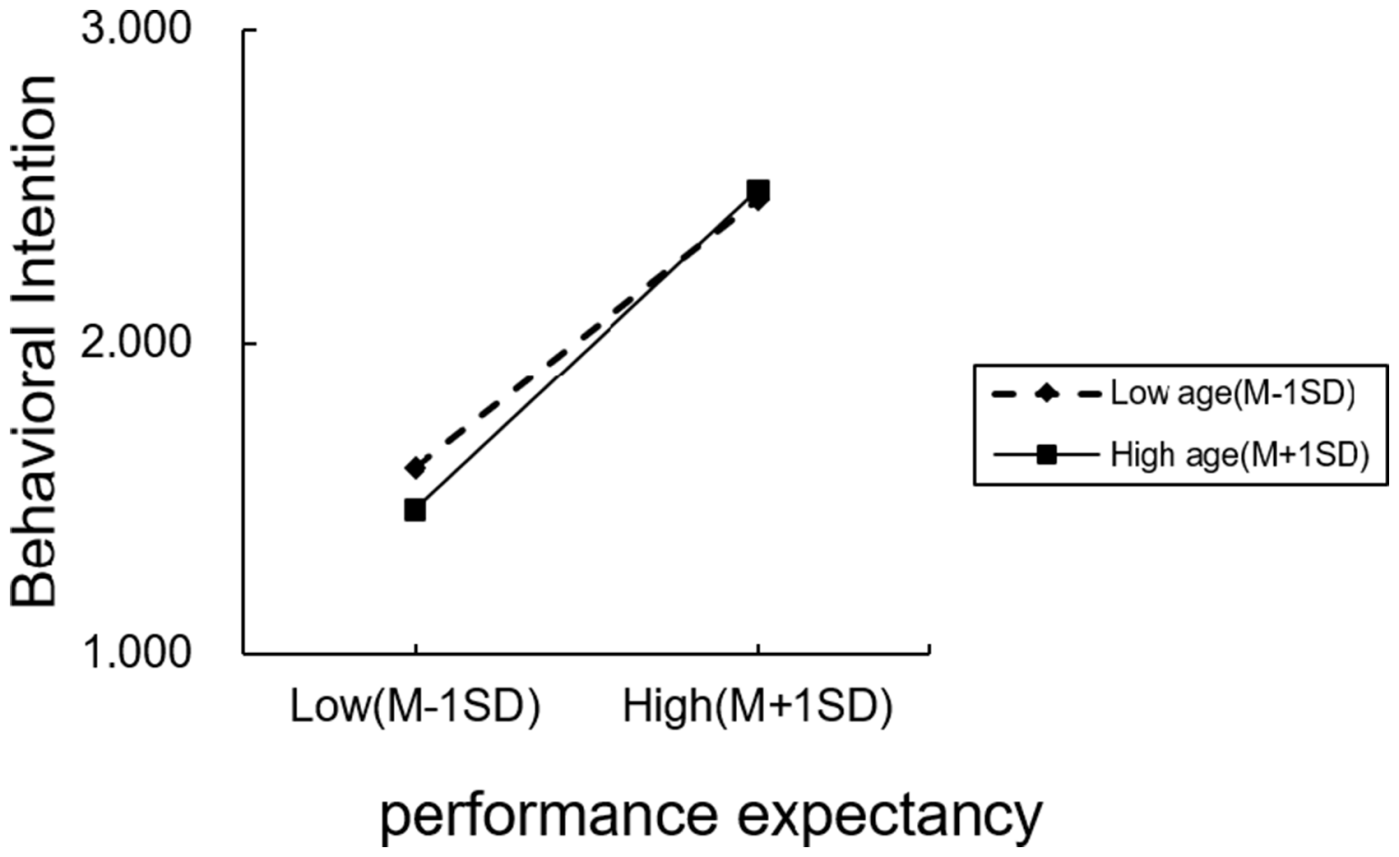
The moderating effect of age on performance expectancy.

This study examines the moderating effects of age on the relationship between performance expectancy, effort expectancy, and behavioral intention. The results indicate that age plays a positive moderating role in the relationship between performance expectancy and behavioral intention (*β* = 0.078, *p* = 0.033), meaning that as age increases, the strength of the effect of performance expectancy on behavioral intention becomes stronger illustrated in Figure 2 (low-age group: *β* = 0.626, *p* < 0.001; middle-age group: *β* = 0.688, *p* < 0.001; high-age group: *β* = 0.749, *p* < 0.001). This indicates that the positive effect of performance expectancy on behavioral intention strengthens as age increases.

**Figure 3:**
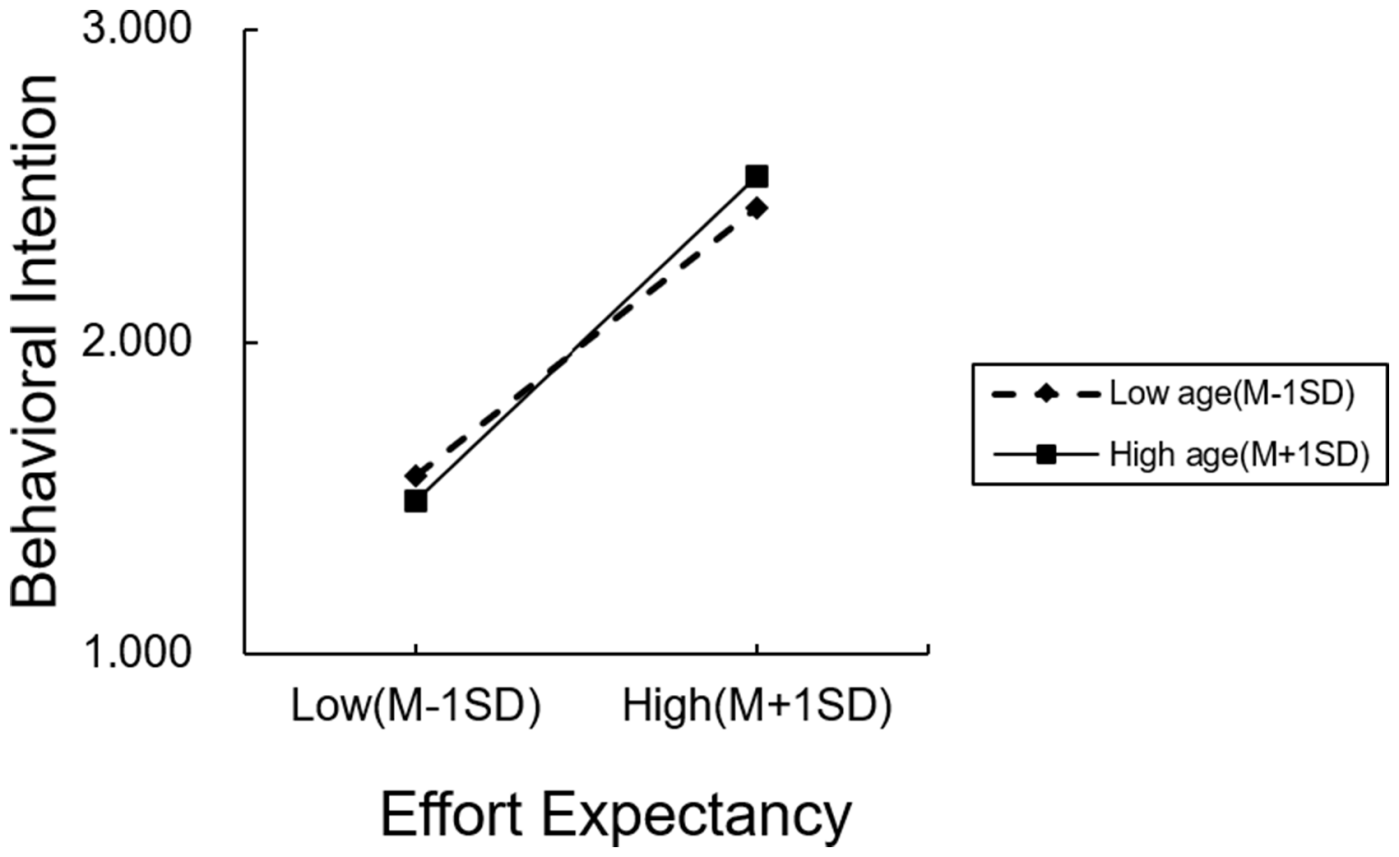
The moderating effect of age on effort expectancy.

**Figure 4:**
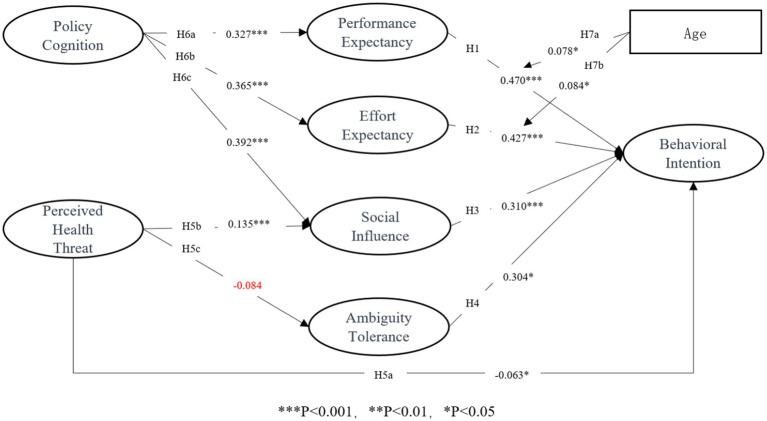
Results of the structural model.

Similarly, age also plays a positive moderating role in the relationship between effort expectancy and behavioral intention (*β* = 0.084, *p* = 0.025). As age increases, the strength of the effect of effort expectancy on behavioral intention becomes stronger illustrated in Figure 3 (low-age group: *β* = 0.636, *p* < 0.001; middle-age group: *β* = 0.702, *p* < 0.001; high-age group: *β* = 0.769, *p* < 0.001). Overall, both performance expectancy and effort expectancy exhibit progressively stronger positive effects on behavioral intention as age increases. The analytical results of this research are depicted in Figure 4.

### 4.3 Multiple-group analysis

After conducting SEM tests and analyzing mediating effects, this research analyzed the demographic groups. The results of the model fit indices for all groups are summarized in Table 8. In terms of the absolute fit indices, the RMR, GFI and AGFI are slightly below the standard values but overall fall within an acceptable range. Regarding incremental fit, the NFI and RFI indices do not meet the criteria, but all the indices are close to the standard values. In terms of parsimonious fit, all indices meet the standard values. Consequently, the various groups in this research exhibited a satisfactory level of model fit.

**Table 8:** Model fitting index.

Fit index		Gender	Marital status	Qualification of drive	Travel distance	Trip destination	Criteria for evaluation
Absolute fit index	CMIN	939.728	918.224	932.777	957.983	950.903	/
	RMR	0.127	0.124	0.130	0.157	0.127	<0.05
	RMSEA	0.049	0.048	0.049	0.047	0.047	<0.08
	GFI	0.866	0.867	0.868	0.872	0.872	>0.09
	AGFI	0.834	0.836	0.836	0.842	0.841	>0.09
Value-added fit index	NFI	0.865	0.867	0.866	0.869	0.870	>0.09
	RFI	0.848	0.846	0.849	0.853	0.853	>0.09
	IFI	0.914	0.916	0.915	0.919	0.919	>0.09
	TLI	0.900	0.905	0.900	0.908	0.909	>0.09
	CFI	0.914	0.915	0.914	0.918	0.919	>0.09
Simple fit index	PGFI	0.677	0.677	0.700	0.704	0.704	>0.05
	CMIN/DF	2.508	2.462	2.488	2.371	2.354	<3.00

The analysis of group invariance was performed using measurement weight models, as shown in Table 8. The chi-square statistic (CMIN) indicates that with an increase in the number of constraints, the measurement weight models for each group change from 10.217 to 15.872 compared to the unconstrained model. The differences in degrees of freedom (DF) are consistent at 13 across all groups. Furthermore, the *p*-values for each group are greater than 0.05, providing evidence that all groups have successfully passed the invariance test.

Before conducting the SEM analysis, various characteristics were grouped. Specifically, gender was used to divide individuals into male and female groups; marital status was based on whether individuals were married, leading to the classification of the sample into married and unmarried groups; and driving qualifications were separated into two groups, those with a driver’s license and those without. Regarding travel distance, this research classified trips within 10 km as short distances and those exceeding 10 km as long distances. The Trip destination were divided into urban and suburban groups. The results of the SEM analysis are presented in Table 9, and a detailed discussion of these findings follows (Table 10).

**Table 9:** Invariance test.

Group	CMIN	D*F*	*R-*value	NFI Delta1	RFI Rho1	IFI Delta2	TLI Rho2
Gender	15.872	13	0.256	−0.002	0.002	0.003	0.003
Marital status	10.217	13	0.676	−0.001	0.004	0.001	0.004
Driving qualifications	12.026	13	0.525	−0.002	0.003	0.001	0.004
Travel distance	10.676	13	0.637	−0.001	0.003	0.002	0.004
Trip destination	13.478	13	0.411	−0.002	0.004	0.002	0.003

**Table 10:** Multi-group analysis.

Group		Influence factors on BI				
		PE	EE	SI	AT	PHT
Gender	Male	0.392***	0.432***	0.325***	0.062	−0.078*
	Female	0.453***	0.470***	0.294***	0.038	−0.051
Marital status	Married	0.450***	0.478***	0.271***	0.064*	−0.026
	Unmarried	0.354**	0.440***	0.382***	−0.003	−0.117**
Driving qualifications	With a driver’s license	0.424***	0.557***	0.235***	0.020	−0.046
	Without a driver’s license	0.406***	0.241	0.487***	0.112	−0.132*
Trip distance	<10 KM	0.288***	0.549***	0.442***	0.027	−0.066
	> = 10 KM	0.479***	0.456***	0.237***	0.060*	−0.061*
Trip destination	Into urban	0.411***	0.450***	0.352***	0.038	−0.041
	Suburban	0.498***	0.459***	0.213**	0.049	−0.141**

BI, behavioral intention; PHT, perceived health threat; PC, policy cognition; SI, social influence; EE, effort expectancy; PE, performance expectancy; AT, ambiguity tolerance. ***p < 0.001, **p < 0.01, *p < 0.05.

Among the gender-specific groups, the male group exhibited a more negative impact of perceived health threat on the intention to use MaaS than the female group (*β* = −0.078, *p* = −0.041). This finding suggests that as the perceived health threat increases among male travelers, their intentions to use MaaS decrease. In contrast, female travelers appear to be less susceptible to the influence of perceived health threat factors.

In terms of marital status, there were significant differences in the path of willingness to use MaaS between the two groups regarding ambiguity tolerance and perceived health threat. Specifically, compared to the unmarried group, the married group showed a greater impact of ambiguity tolerance on the willingness to use MaaS (*β* = 0.064, *p* = 0.015). Unlike in the married group, in the unmarried group, the perceived health threat negatively impacted the willingness to use MaaS (*β* = −0.117, *p* = −0.002). As the perception of health threats increases among unmarried travelers, their willingness to use MaaS decreases. However, enhancing ambiguity tolerance significantly increases the willingness of married travelers to use MaaS.

Overall, the driver qualification characteristics exhibited significant differences between the two groups in terms of the influence of effort expectancy and perceived health threat on the intention to use MaaS. Compared to the group without a driver’s license, the group with a driver’s license showed a greater impact of effort expectancy on the intention to use MaaS (*β* = 0.557, *p* = 0.000). In contrast, a lack of a driver’s license negatively impacted the intention to use MaaS (*β* = −0.132, *p* = 0.013). Consequently, it is evident that licensed travelers are more concerned about the perceived difficulty of using MaaS, while an increase in perceived health threats among non-licensed travelers leads to a decrease in their intentions to use MaaS.

In the context of travel distance characteristics, compared to those in the short-distance group, there were significant differences in the paths of perceived health threat (*β* = −0.061, *p* = 0.047) and ambiguity tolerance (*β* = 0.060, *p* = 0.029) on the intention to use MaaS within the long-distance group. It can be inferred that for long-distance travelers, an increase in ambiguity tolerance increases their intentions to use MaaS, while perceived health threats significantly decrease their intentions to use MaaS.

Among the two travel destination groups, the suburban group showed a significantly negative impact of perceived health threat factors on the intention to use MaaS (*β* = −0.141, *p* = 0.010). This implies that as the degree of perceived health threat increases, travelers with suburban destinations show lower inclinations to use MaaS.

## 5 Discussion

This research investigates MaaS based on the extended UTAUT model to elucidate the process of travelers accepting MaaS during significant public health events. In addition to the three existing influencing factors in the model, we introduce psychological factors such as ambiguity tolerance, perceived health threat, and policy cognition, thus enhancing the explanatory power of the original model. In the following sections, we provide a detailed analysis of the research findings, explore connections with the literature on MaaS, and subsequently engage in separate discussions.

### 5.1 UTAUT-related variables

Consistent with the findings of previous research, this research verified that travelers’ intentions to use MaaS are influenced by their performance expectancy and social influence factors (van’t Veer et al., 2023; Ye et al., 2020). However, Madigan et al. (2017) argued that effort expectancy does not significantly affect usage intention. In contrast, our research comes to a different conclusion. The reason lies in the fact that MaaS, being simple and user friendly, can better meet travelers’ expectations for using the service, thus significantly influencing their intentions to use MaaS. This study further concludes that age plays a moderating role in the relationships between performance expectancy, effort expectancy, and behavioral intention. Specifically, the positive influence of both performance expectancy and effort expectancy on behavioral intention strengthens as age increases, demonstrating that older travelers tend to place greater importance on these factors when considering the use of MaaS. Additionally, our research reveals that effort expectancy is not a significant factor considered by non-qualified drivers when considering the use of MaaS. This is because travelers with driving qualifications or experience can compare the ease of use of MaaS with their past experiences of self-driving, increasing susceptibility to the influence of this factor.

### 5.2 Ambiguity tolerance

Individual ambiguity tolerance affects behavioral decision-making, confirming research findings that ambiguity tolerance influences individual behavioral decisions (Hazen et al., 2012; Tian and Li, 2015). Building on previous research, this research identifies a significant positive impact of travelers’ ambiguity tolerance on their intentions to use MaaS. This finding suggests that travelers with greater ambiguity tolerance can mitigate the impact of unfamiliar information introduced by MaaS. In contrast, travelers with lower ambiguity tolerance may need to invest substantial time and effort in learning how to navigate various package options and travel routes, adapting to this new technology and consequently decreasing their intentions to use MaaS. Demographic analysis of population characteristics revealed that the level of ambiguity tolerance in married individuals and those engaged in long-distance travel is positively correlated with their intentions to use MaaS.

### 5.3 Perceived health threat

This research also revealed that perceived health threat factors negatively impact individual behavioral decisions (Kapser et al., 2021). This influence is particularly pronounced for unmarried males and non-driving-eligible travelers. Additionally, when individuals travel longer distances and their destinations are concentrated in suburban areas, perceived health threats become a significant factor leading to a decrease in their willingness to use MaaS. Based on this, the research revealed that individual perceived health threat factors indirectly affect individuals’ intentions to use MaaS through the mediation of social influence factors. The rationale behind this lies in the gradual increase in the perceived level of health threat, which prompts people to become more sensitive to health and safety-related information (Hadjistavropoulos et al., 2012). Travelers may perceive MaaS-related information provided by significant others as a protective mechanism against health threats. Consequently, increased perceptions of health threats lead travelers to exhibit positive attitudes and intentions toward MaaS, thus increasing their willingness to use MaaS.

Unlike expected outcomes, perceived health threats do not influence travelers’ adoption of MaaS through ambiguity tolerance. The reason for this divergence might be because the fact that the ambiguous information encountered by travelers in the context of using MaaS arises primarily from processes such as software use and package selection. In this scenario, it becomes difficult for the perceived health threat to exert its impact. As a result, the research yielded statistically insignificant findings.

### 5.4 Policy cognition

Previous research has predominantly analyzed the impact of individual policy cognition on policy acceptance (Luo M. et al., 2022; Luo P. et al., 2022; Wang et al., 2020). In contrast, this research examines the potential influence of policy cognition factors on individual behavioral decision-making. The findings reveal the significant role of policy cognition factors in shaping the intention of travelers to use MaaS. Furthermore, the research revealed that policy cognition factors positively influence travelers through three core factors: performance expectancy, effort expectancy, and social influence. This implies that the formation and communication of policy cognition are not only processes of information transmission but also forces the directly shape the attitudes and behaviors of travelers. As a result, this promotes positive engagement and the intention to use MaaS among travelers.

### 5.5 Contribution and policy implications

The theoretical contributions of this research are multifaceted. First, based on previous research, we extend the research context of the UTAUT model. We elucidated the changes in travelers’ willingness to use MaaS during significant public health events. This expansion improves the generalizability of the UTAUT theoretical model in the domain of MaaS and public health event research. Second, our research innovatively analyses the impact of travelers’ tolerance of ambiguity on individual usage intentions in the MaaS research domain, thus supplementing existing research findings. Third, while existing research have examined the mechanisms through which perceived health threats influence individual decision-making behavior, our research focuses specifically on the MaaS research domain, thus enhancing the explanatory power of relevant theories. Finally, unlike previous research, our research reveals the impact of restrictive travel policies on travelers’ use of MaaS, providing theoretical support for the influence of policy cognition on individual decision-making behavior.

Based on the conclusions drawn from the aforementioned research, this research proposes the following policy recommendations. First, from a corporate perspective, the government should encourage the integration of various types of mobility service platforms and foster collaboration and breakdown barriers to create a favorable environment for the development of MaaS. For example, in Beijing, since November 2019,^1^ Beijing has launched the MaaS 1.0 platform, integrating services from Amap and Baidu Maps to provide features such as real-time bus updates and subway congestion information, improving the travel experience for multimodal trips like “public transit + walking.” In September 2020, the platform introduced a carbon reduction incentive mechanism, allowing users to earn carbon credits through green travel methods (e.g., public transit, walking, and cycling), which could be redeemed for transit cards, vouchers, or donated to environmental programs. Over 3 years, the platform has attracted over 30 million users, enabling 4.5 million green trips daily and achieving nearly 400,000 tons of cumulative carbon reduction through its 3.54 million registered users.

Second, from the perspective of travelers, the government can incentivize individuals to use MaaS for their commuting needs, reduce the frequency of private car use, and enhance road capacity. Furthermore, during major public health emergencies, the government’s assessment of the level of travel can effectively reduce perceived health threats among travelers, helping alleviate concerns during the commuting process. Finally, after the event, the government’s communication efforts regarding restrictive travel policies contributed to raising awareness and understanding of such policies among travelers, thereby increasing their trust in the adopted modes of transportation.

## 6 Conclusion

Given the potential of MaaS to alleviate traffic congestion and enhance travel experience, this research investigates travelers’ intentions to use MaaS. Based on an adapted UTAUT model, this research analyses the attitudes of travelers toward MaaS during a major public health crisis outbreak. The research findings reveal that the three original UTAUT factors and ambiguity tolerance all exert positive influences on the intentions of travelers to use MaaS. In addition to directly and negatively impacting the intention to use, perceived health threats indirectly affect travelers’ willingness to use MaaS through the mediating factor of social influence. Conversely, policy cognition, on the other hand, indirectly influences travelers’ intentions to use MaaS through the factors of performance expectancy, effort expectancy, and social influence.

## 7 Limitations and outlook

Future studies should build upon this preliminary research by incorporating larger and more geographically diverse samples, as well as integrating various data collection methods. In the future research, in conjunction with field surveys or experimental designs, should explore the specific manifestations of ambiguity tolerance in other contexts and its specific impact pathways on the intention to use MaaS. Although this research explored the impact of restrictive travel policies on travelers’ use of MaaS, it may not have fully and objectively considered the influences of various policies. Future research should conduct cross-regional and cross-national comparative studies to gain a deeper understanding of the effects of different policy backgrounds, thus providing more precise recommendations for practical policy formulation.

## Data Availability

The original contributions presented in the study are included in the article/supplementary material, further inquiries can be directed to the corresponding author.

## Footnotes

^1^Beijing Municipal Commission of Transport. Beijing’s MaaS Mobility Platform to be Upgraded; New Energy Vehicles to Receive Carbon Incentives. Beijing Municipal People’s Government Portal. Retrieved December 3, 2024, from https://www.beijing.gov.cn/ywdt/gzdt/202306/t20230630_3150638.html
